# Computational analysis of network activity and spatial reach of sharp wave-ripples

**DOI:** 10.1371/journal.pone.0184542

**Published:** 2017-09-15

**Authors:** Sadullah Canakci, Muhammed Faruk Toy, Ahmet Fatih Inci, Xin Liu, Duygu Kuzum

**Affiliations:** 1 Electrical and Computer Engineering Department, Boston University, Boston, Massachusetts, United States of America; 2 Electrical and Computer Engineering Department, University of California San Diego, La Jolla, California, United States of America; 3 Electrical and Electronics Engineering, Middle East Technical University, Ankara, Turkey; 4 Faculty of Engineering and Natural Sciences, Sabanci University, Istanbul, Turkey; Consejo Superior de Investigaciones Cientificas, SPAIN

## Abstract

Network oscillations of different frequencies, durations and amplitudes are hypothesized to coordinate information processing and transfer across brain areas. Among these oscillations, hippocampal sharp wave-ripple complexes (SPW-Rs) are one of the most prominent. SPW-Rs occurring in the hippocampus are suggested to play essential roles in memory consolidation as well as information transfer to the neocortex. To-date, most of the knowledge about SPW-Rs comes from experimental studies averaging responses from neuronal populations monitored by conventional microelectrodes. In this work, we investigate spatiotemporal characteristics of SPW-Rs and how microelectrode size and distance influence SPW-R recordings using a biophysical model of hippocampus. We also explore contributions from neuronal spikes and synaptic potentials to SPW-Rs based on two different types of network activity. Our study suggests that neuronal spikes from pyramidal cells contribute significantly to ripples while high amplitude sharp waves mainly arise from synaptic activity. Our simulations on spatial reach of SPW-Rs show that the amplitudes of sharp waves and ripples exhibit a steep decrease with distance from the network and this effect is more prominent for smaller area electrodes. Furthermore, the amplitude of the signal decreases strongly with increasing electrode surface area as a result of averaging. The relative decrease is more pronounced when the recording electrode is closer to the source of the activity. Through simulations of field potentials across a high-density microelectrode array, we demonstrate the importance of finding the ideal spatial resolution for capturing SPW-Rs with great sensitivity. Our work provides insights on contributions from spikes and synaptic potentials to SPW-Rs and describes the effect of measurement configuration on LFPs to guide experimental studies towards improved SPW-R recordings.

## Introduction

Hippocampal network oscillations have been extensively investigated due to their potential roles in learning, memory, spatial navigation, and consolidation of memories [1–5]. The anatomically well-defined structure of the hippocampus has enabled numerous neuroscience studies over decades. Three major network oscillations generated in hippocampus, theta (6–10 Hz), gamma (30–120), and SPW-Rs (150–250 Hz), are hypothesized to participate in memory formation and consolidation [2]. Among these, SPW-Rs are the most synchronous pattern in the mammalian brain [2]. They occur in the hippocampus during slow wave sleep, immobility, and consummatory behaviors [6]. SPW-Rs are composed of high amplitude sharp waves and high frequency ripple oscillations. Recent studies have shown that during SPW-Rs, firing patterns of sequentially activated place cells, observed during wakeful exploration, are replayed in forward or reverse order [7–10]. Online disruption of SPW-Rs has been shown to cause memory impairment [11,12], indicating SPW-Rs’ role in memory consolidation. SPW-R replay has also been suggested to play important roles in combining recently acquired and pre-existing information to influence decisions, plan actions, and potentially allow for creative thoughts [2].

Understanding the neural processes and physiological roles of different hippocampal regions during SPW-Rs generation is crucial towards deciphering the mechanisms for replay and memory consolidation. A model explaining SPW-R generation in the hippocampus has been proposed by Buzsaki et al. [13,14]. According to this model, SPW-Rs arise from the excitatory recurrent system of the CA3 region. The recurrent connectivity in the CA3 region serves as a perfect template for synchronous bursting of CA3 neurons resulting in the generation of high amplitude sharp waves, which form the low frequency component of the SPW-Rs. Sharp waves traveling to CA1 induce excitation, bringing about a fast network oscillation (ripples) in CA1. This hypothesized model considers physiological roles of different hippocampal regions to elucidate the generating of SPW-Rs. Several different mechanisms have been proposed for generating sharp waves and ripples, separately. Sharp waves emerging from the excitatory recurrent CA3 network was modeled by Traub and Wong [15], using 100 CA3 compartmental neurons, each capable of intrinsic bursting and randomly interconnected by excitatory chemical synapses. Taxidis et al. [16,17] extended the Traub model to a one-dimensional array of 1,000 pyramidal cells and 100 interneurons. In the Taxidis model, spike bursts were initiated in CA3, producing a corresponding burst of activity in CA1 by exciting both pyramidal cells and interneurons through the Schaffer collaterals. On the ripple side, three different mechanisms have been proposed. The first mechanism involves gap junctions between pyramidal cells, providing strong coupling for synchronous firing as a result of input arriving from CA3 [18–20]. The second mechanism proposes reciprocal inhibition between CA1 interneurons and pyramidal cells pacing the spiking activity of pyramidal neurons at the ripple frequency [13,14,16]. The third mechanism suggests that feedback inhibition between interneurons and pyramidal cells [13,21,22] generates ripples as a result of external input to both cells. More recently, the combination of reciprocal and feedback inhibition between the pyramidal cells and the inhibitory network has also been suggested as a potential mechanism for ripple generation [23].

While different models successfully described certain characteristics of experimental SPW-Rs [15–17,24–26], they did not investigate the experimentally observed distributions of SPW-R amplitude or spatiotemporal characteristics of local field potentials (LFPs). In addition, understanding specific contributions from neuronal spikes or synaptic potentials to SPW-Rs remains to be addressed.

In this work, we investigated how measured LFPs are related to neuronal spikes and synaptic potentials during SPW-Rs and how microelectrode distance and area affect SPW-R recordings using a previously developed biophysical model [25–27]. First, we performed computational analysis to understand contributions from synaptic potentials and neuronal spikes to SPW-Rs by computing LFPs generated by the CA1 network for two different mechanisms. Then we investigated the spatial extent of LFPs and how LFP amplitudes scale with distance from the source of the activity. We studied the effects of spatial averaging on measured SPW-R characteristics for different electrode sizes. We simulated LFPs recorded with a high-density microelectrode array spanning an area comparable to the neuronal network (4 × 4 mm^2^). Our results suggest that pyramidal cell spiking is essential for generation of SPW-Rs. Ripple-like oscillations generated only by synaptic potentials have much lower amplitude and are predicted to be below experimentally measurable limits. Furthermore, we demonstrate that amplitudes of ripples exhibit a steep decrease with increasing distance for spatially confined ripples. High frequency oscillations at 200 Hz or higher can only be detected if the electrode is located in close proximity to the network generating ripple activity. Finally, our results suggest that the electrode area is a critical parameter, which is directly related to measurement range. The relative amplitudes of sharp waves and ripples notably decrease with larger electrode sizes due to spatial averaging and distance scaling, and the decrease is more prominent when the recording electrode is closer to the network generating the signals. As a result, the electrode size can produce substantial differences in the LFP measurements. Our findings indicate the importance of choosing the right measurement configurations and finding the ideal spatial resolution for capturing microcircuit activities in experimental studies.

## Results

### Biophysical model

**Fig 1:**
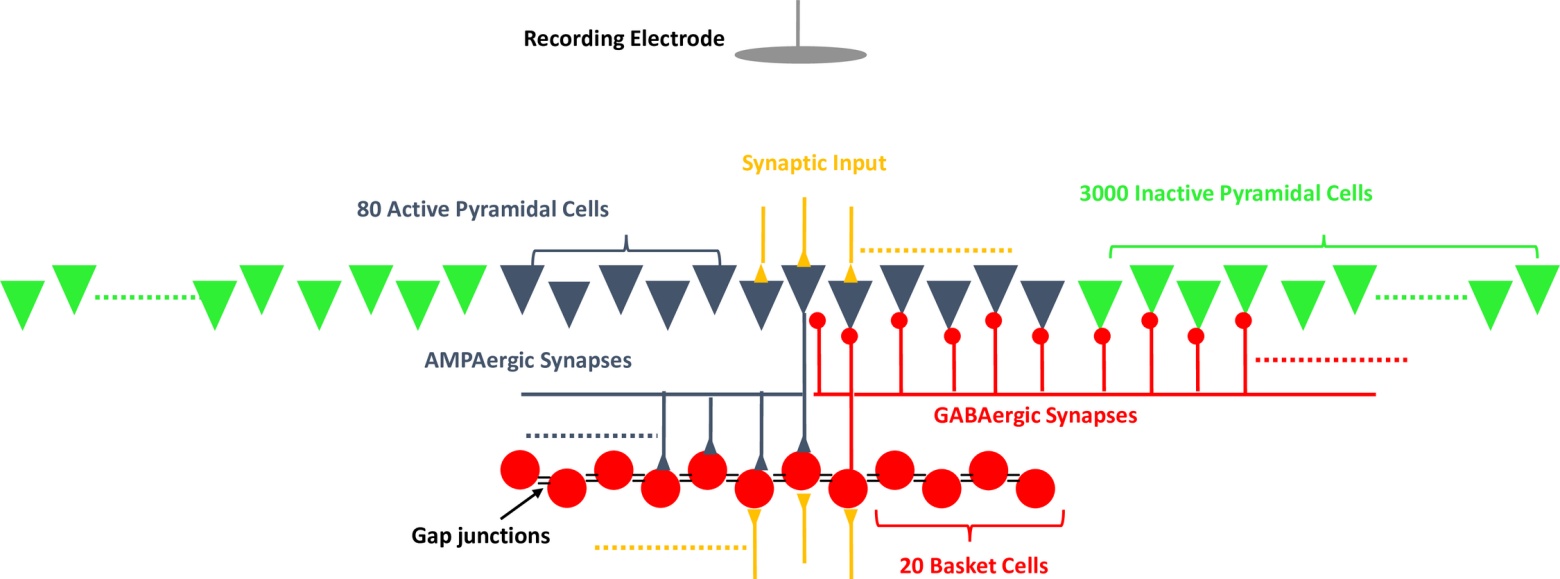
Schematic of the computational model of hippocampus. The model consists of 3080 pyramidal cells, with only 80 of them actively spiking, and 20 basket cells. Both 80 active pyramidal cells and 20 basket cells have excitatory synaptic input. Basket cells make gap junctions to the nearest basket cells. They send GABAergic synapses to pyramidal cells and receive feedback AMPAergic synapses from active basket cells. The total network spans an area of 1 × 1 mm^2^. Activated pyramidal cells and basket cells are distributed across a 2D plane with an area of 400 × 400 μm^2^ while the inactive pyramidal cells cells are distributed across an area of 1 × 1 mm^2^. The schematic shows the side view of the 2D array to clarify the synaptic connectivity. Recording electrodes of various sizes are placed at varying distances from the network.

We used a biophysical model of CA1 to investigate the network properties capable of generating SPW-Rs. The model is based on previously published models implemented on the NEURON 7.3 platform [25–27] developed by William Stacey’s group. The model is capable of generating a broad spectrum of hippocampal high frequency oscillations with different frequencies and durations consistent with recordings in *in vivo* and *in vitro* studies [26]. It follows a generalized approach of using a single network with inhibitory feedback between pyramidal and basket cells, consistent with the pyramidal layer of CA1 region, where ripples are measured experimentally. The ratio of the active basket and pyramidal cells is chosen as 1:4 to be consistent with CA1 anatomy [28]. The 2D model consists of 80 pyramidal cells and 20 basket cells (Fig 1) as the core network. Eighty pyramidal cells actively driven by excitatory synapses represent a small cluster of active cells within a large network of 3100 neurons. The remaining 3000 pyramidal cells are inactive and do not spike since they do not receive any excitatory input. They serve as neighboring networks that only generate synaptic potentials. This physiologically realistic 2D model of CA1 allows exploration of various spatial network effects on LFPs and enables detailed analysis of spatiotemporal characteristics of LFPs across a 2D network, which is different from previously developed 1D models or models with no specific network structure. In the model, each pyramidal cell has five cylindrical compartments: a soma with a diameter of 20 μm, and a length of 20 μm, a basal dendrite with a diameter of 2 μm, and a length of 200 μm, and three apical dendrites each with diameters of 2 μm, and lengths of 150 μm. Compartments are located end to end on the axis perpendicular to the 2D plane of the network and their order is basal dendrite, soma, and apical dendrites, as shown from top to bottom in Fig 1. There are 20 basket cells for inhibition, which consist of three-compartment soma with a diameter of 10 μm, and a length of 3.18 μm, each. The recording electrode is placed above the 2D plane of pyramidal cells on the side of the basal dendrites close to the somas of the pyramidal cells (stratum oriens and stratum pyramidale layers) as shown in Fig 1. The basic connectivity of the model consists only of the inhibitory feedback between pyramidal and basket cells. As shown in Fig 1, basket cells send GABAergic synapses to all pyramidal cells (τ_rise_ = 1.5ms, τ_decay_ = 8.0 ms, g_max_ = 5.5 nS, E_rev_ = -80 mV) and receive AMPAergic synapses as feedback from only 10 activated pyramidal cells (τ_rise_ = 0.2 ms, τ_decay_ = 1.0 ms, gmax = 0.5 mS/cm^2^, E_rev_ = 0 mV). In addition to these connections, basket cells are coupled to each other with somatic gap junctions to the nearest basket cell. As for the activated pyramidal cells, each has efferent AMPAergic synapses with approximately 2 to 3 basket cells. Since the remaining 3000 pyramidal cells do not receive excitatory input and only receive inhibitory input, they do not fire action potentials (APs). Therefore, the main purpose of these cells in the model is to understand the effects of synaptic potentials from basket cells. The total network spans an area of 1 × 1 mm^2^. Activated pyramidal cells and basket cells enclose an area of 400 × 400 μm^2^ while the inactive pyramidal cells span a 1 × 1 mm^2^ area. NEURON code for the model is available in ModelDB [29] with accession number 230861.

In our model the driving input for the network is excitation by afferent synaptic activity. The synaptic input to basket and pyramidal cells is modulated by changing the intensity of AMPA synaptic noise, as described in previous publications [25–27]. The synaptic input is a Poisson process, where the mean of the distribution determines the noise intensity. It has been previously shown [25–27] that modulating synaptic input can produce high frequency oscillations at different frequency bands. In this model, we set the synaptic noise to generate high frequency oscillations at 200 Hz to simulate SPW-Rs.

**Fig 2:**
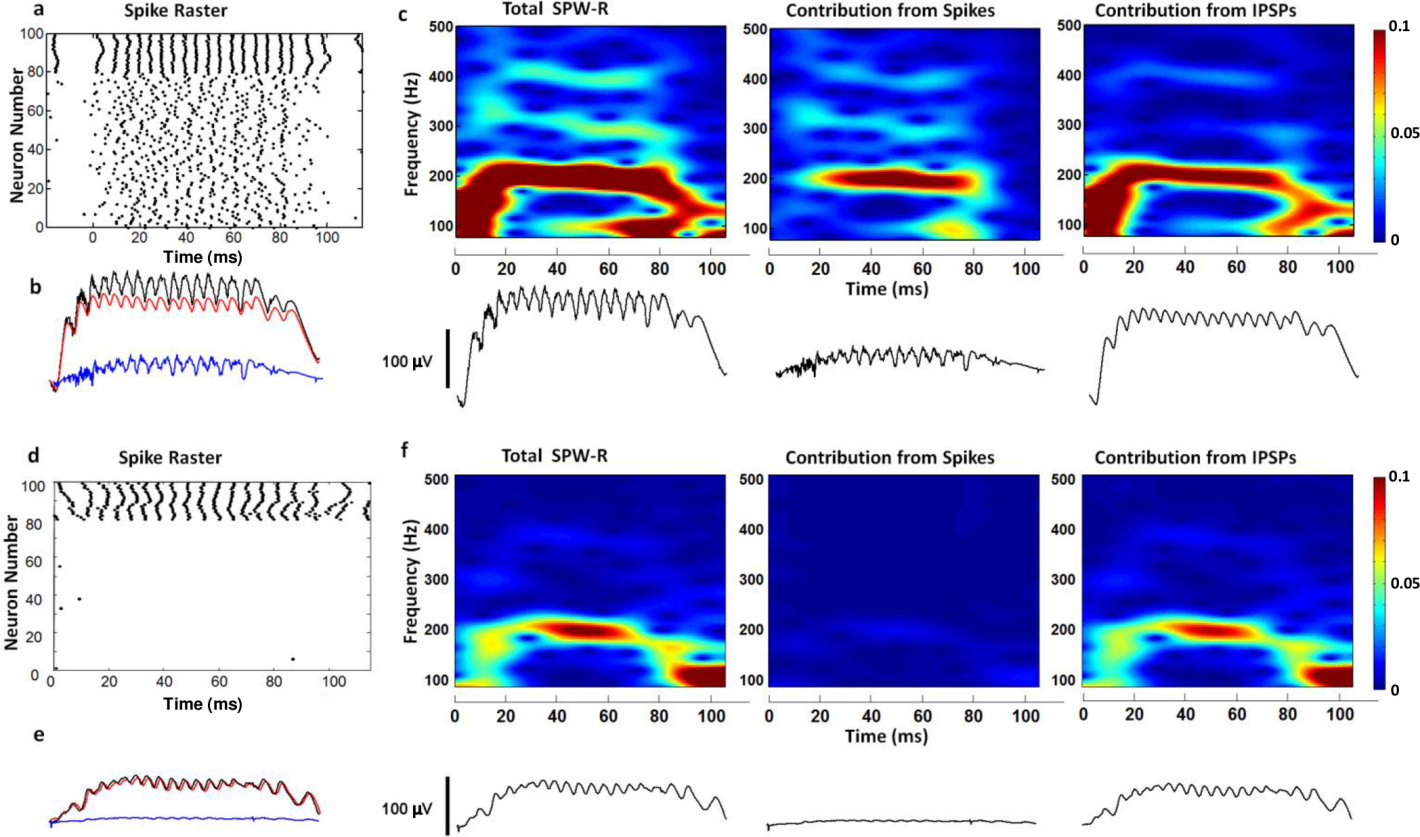
Synaptic potentials and neuronal spikes contributions to SPW-Rs. **(a)** Spike raster for 80 active pyramidal cells (0–80) and basket cells (80–100) showing synchronous firing (for a noise intensity of 0.77 nA^2^). Spike raster does not show 3000 non-firing pyramidal cells. (**b)** LFP waveforms for total SPW-R (black), contributions from IPSPs (red), and contributions from spikes (blue) are shown. **(c)** Spectrograms are shown for total SPW-Rs (left), spikes (center) and IPSPs (right). IPSPs are responsible for high amplitude sharp waves (c, right). Spikes from individual neurons only contribute to ripples (c, center) at 200 Hz and weaker contributions at 300 Hz and 400 Hz. **(d)** Spike raster showing only basket cell firing (for a noise intensity of 2.5 × 10^−4^ nA^2^). **(e**) LFP waveforms for total SPW-R (black), contributions from IPSPs (red), and contributions from spikes (blue) are shown. IPSPs are the main contributor to SPW-Rs. **(f)** Spectrograms are shown for total SPW-Rs (left), spikes (center) and IPSPs (right).

LFP signals are simulated considering all transmembrane and postsynaptic currents from various compartments of cells. Spatial density and synchrony of the transmembrane and postsynaptic currents affect the LFP waveform (Fig 2). The most important characteristic features of the LFP signals, such as amplitude and frequency, depend on these contributions stemming from various current sources. Therefore, by recording transmembrane current for all N compartments [30], we have computed the net electric potential at the recording electrode using the source-field model of current monopoles:

$$\Phi (r,t)=\frac{1}{4\pi \sigma }\sum_{n=1}^{N}\frac{I_{n}(t)}{\left|r-r_{n}\right|}$$ (1)

where Φ (r, t) is the total electrode potential at time t, σ is the extracellular conductivity, n is the compartment number, I_n_ is the transmembrane current generated from compartment n, and |r-r_n_| is the distance between compartment n and the recording electrode. Our simulated LFP signals capture the main components of experimentally observed SPW-Rs including high amplitude sharp waves combined with ~200 Hz ripple oscillations (Fig 2).

In experimental studies, SPW-Rs are detected by metal microelectrodes, which have a finite conductive recording surface. Finite-sized electrodes have been suggested to measure the average potential across the electrode surface [31–33]. Therefore, the potential recorded by a finite-sized electrode can be approximated as the average potential across its surface S:

$$\Phi (r,t;S)=\frac{1}{A_{S}}\iint_{S}\Phi (r^{'},t)d^{2}r^{'}\approx \frac{1}{m}\sum_{i=1}^{m}\Phi_{i}(r_{i}^{'},t)$$ (2)

Here, m represents the number of point electrodes inside the flat surface. In our model, a mesh structure consisting of a periodic array of point electrodes with 10 μm spacing is formed to calculate the field potentials across the 2D space, similar to the methodology used in finite element modeling. LFPs are computed at every point of the mesh and integrated across the surface of the finite size electrode. For comparative analyses with experimental studies, field potentials are computed by five different sizes of electrodes (100 × 100 μm^2^, 300 × 300 μm^2^, 1 × 1 mm^2^, 2 × 2 mm^2^, 4 × 4 mm^2^). It is important to mention that our calculations provide an approximate solution for LFPs recorded across a surface [34]. It takes into account the averaging effect of the metal electrode surface [31–33], while not including higher order electric field or edge effects, depending on electrode geometry. A more complete and detailed treatment could utilize frequency-dependent finite element models (FEM) for the microelectrodes [35] coupled with the neuronal network model implemented in the NEURON 7.3 platform. However, due to the large size of the network and the large number of grid points in 3D space between the neural population and the neural recording electrodes, FEM approach would be computationally demanding.

### Contributions from synaptic potentials and neuronal spikes to SPW-Rs

Previous studies have suggested that CA1 ripples emerge from local mechanisms rather than being transferred from upstream regions in the hippocampus [1,36,37]. Recent work [23] using optogenetic stimulation has shown that local activation of interneurons and pyramidal cells in CA1 can lead to the emergence of high frequency oscillations, while optogenetic activation of only interneurons in CA1 is not sufficient by itself to induce measurable oscillations. These experimental findings suggest that pyramidal neuron activity is necessary for ripple generation. In order to understand relative contributions from the spiking of pyramidal neurons and synaptic potentials generated by interneurons, we performed simulations using two different mechanisms for SPW-R generation: synchronous pyramidal cell spiking (Fig 2A–2C) and synchronous IPSPs induced in pyramidal cells (Fig 2D–2F). In order to maintain these conditions in simulations, we changed the synaptic input parameters to the active pyramidal cells and the basket cells. Spectrograms are used to understand different characteristic features of these two separate mechanisms. In Fig 2A–2D, the 80 active pyramidal cells and 20 basket cells are included in raster but the remaining 3000 pyramidal cells are not included because they do not fire action potentials and only contribute synaptic currents.

In the first SPW-R generation mechanism, which is synchronous pyramidal cell spiking, a sparse population of pyramidal cells (~3%) generates APs to reveal synchronous spiking activity (Fig 2A). The raster plot in Fig 2A shows that pyramidal cells are firing along with synchronous firing of basket cells. Our simulation results reveal that the high amplitude sharp wave component of SPW-Rs is originated from synaptic activity (Fig 2C, right most), mainly driven by spiking activity of the basket cells. Synaptic activity also contributes to the ripples at 200 Hz. Spikes (APs) from pyramidal cells contribute to ripples (Fig 2C, center) at 200 Hz and weaker contributions at 300 Hz and 400 Hz. Contributions of spikes do not affect the amplitude of the sharp wave (Fig 2) significantly. Contributions from each cell compartment are shown separately for the synchronous pyramidal cell spiking case in the Supplementary information (S1 Fig). We have also investigated synchronous IPSPs as a potential mechanism for generation of SPW-Rs or ripples. As shown in Fig 2D–2F, SPW-R-like oscillations can be generated without any pyramidal cell firing. For this second mechanism, only baskets cells fire APs inducing IPSPs in all of the pyramidal cells [13,14,38], which give rise to ripples at 200 Hz. Spectrograms in Fig 2F indicate that all the contributions to SPW-Rs for the second mechanism are originated by IPSPs. Although the generated waveform resembles SPW-Rs, the amplitudes of both sharp waves and ripples are much smaller compared to the pyramidal cell spiking case. Especially, the amplitude of the ripples seems to be below experimentally measurable limits as shown in the Supplementary information (S2 Fig). In conclusion, our findings indicate that pyramidal cell spiking is an essential mechanism for generation of experimentally-measurable SPW-Rs, consistent with recent experimental work by Stark et al. [23] and computational modeling work by Malerba et al. [24].

**Fig 3:**
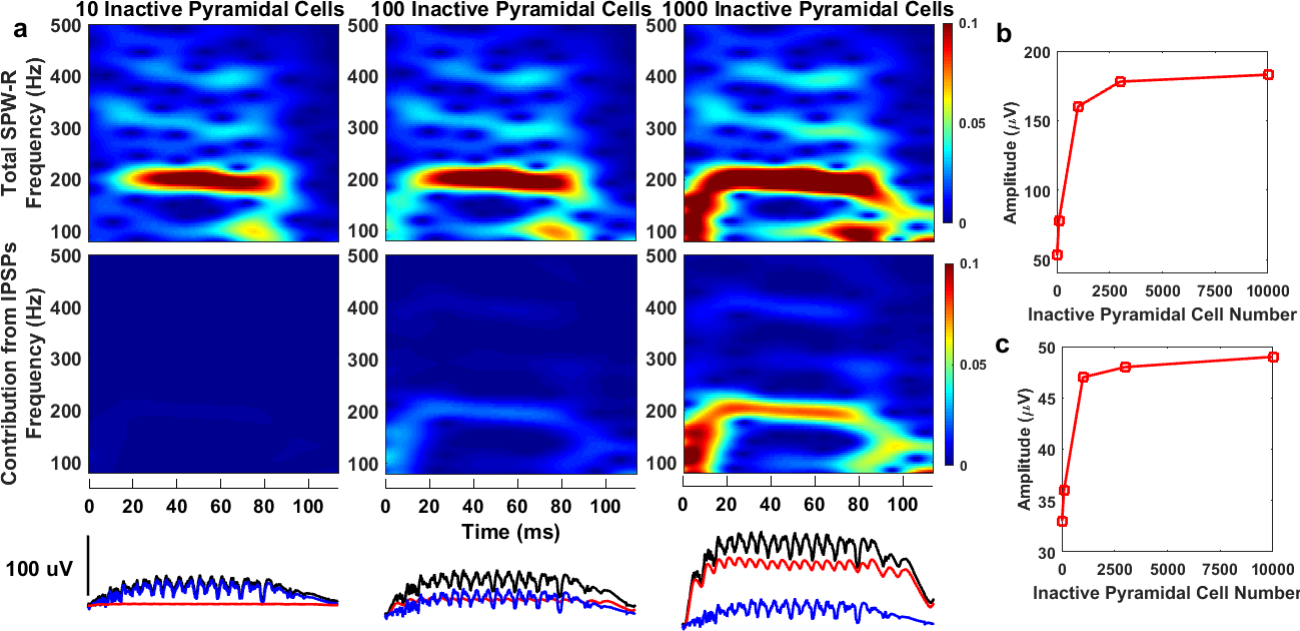
Effect of number of inactive pyramidal cells on SPW-Rs. **(a) S**pectrograms of total SPW-Rs and contribution from IPSPs and LFP waveforms for total SPW-Rs (black), contributions of spikes (blue), and contributions of IPSPs (red) for 10, 100, and 1000 inactive pyramidal cells. (**b-c)** Graphs of the amplitudes of sharp waves (b) and ripples (c) vs. inactive pyramidal cell number.

Experimental studies suggest that some of the SPW-R events recorded in CA1 are generated locally by a small network, and they remain local and confined to specific CA1 segments [1]. We investigated the contribution from IPSPs to SPW-Rs in such a scenario by changing the number of inactive pyramidal cells, which serve as a synaptic potential contributor in the network. Since they do not receive any excitatory input, but instead receive inhibitory input from the basket cells, they only contribute to IPSPs. We varied the number of inactive pyramidal cells in the range of 0 to 10000, while keeping constant the noise intensities to active pyramidal cells and basket cells, and the numbers of active pyramidal cells and basket cells and the distance between the inactive pyramidal cells. Therefore, the spike rasters and the contribution of spikes to SPW-Rs do not change and only the contribution of IPSPs to SPW-Rs depends on the inactive pyramidal cell number. Fig 3 shows spectrograms and SPW-R waveforms for 10, 100, and 1000 inactive pyramidal cells (3000 and 10000 are included in Supplementary information, S3 Fig). As expected, the frequency spectrum of SPW-Rs does not exhibit significant changes with the inactive pyramidal cell number. However, the amplitudes of the sharp waves (Fig 3B) and the ripples (Fig 3C) increase with the increasing number of inactive pyramidal cells. The increase in amplitude shows a steep trend up to 1000 cells and exhibits a more gradual increase as the inactive pyramidal cell number is further increased. Increasing the number of inactive pyramidal cells while keeping the distance (neuron density in the network) between the cells constant increases network area. For larger area networks, contributions to the LFP from cells far away from the measurement point are weaker. Therefore, increasing the cell number does not have a huge impact on SPW-Rs after a certain number of cells in the network. These findings are consistent with experimental recordings where LFPs show contributions from cells at certain distances from the electrode [39].

### Effect of distance on SPW-Rs

Understanding the spatial extent of ripples locally generated in CA1 networks is crucial to gain insights on SPW-R propagation across hippocampal circuits. *In vivo* recordings investigating ripple generation and propagation [40] have suggested that the amplitude of local ripples exhibits strong attenuation with distance and decreases 10% of peak value over a 1 mm distance. In order to understand spatial extent and amplitude scaling of ripples generated by spatially confined small populations in CA1, we simulated the effects of different recording electrode distances on the amplitude of LFPs. LFPs at six different distances (0 μm, 25 μm, 50 μm, 75 μm, 150 μm, 300 μm) from the active network and three different recording areas, including an ideal point electrode, and electrodes with surface areas of 300 × 300 μm^2^, and 1 × 1 mm^2^ were computed.

**Fig 4:**
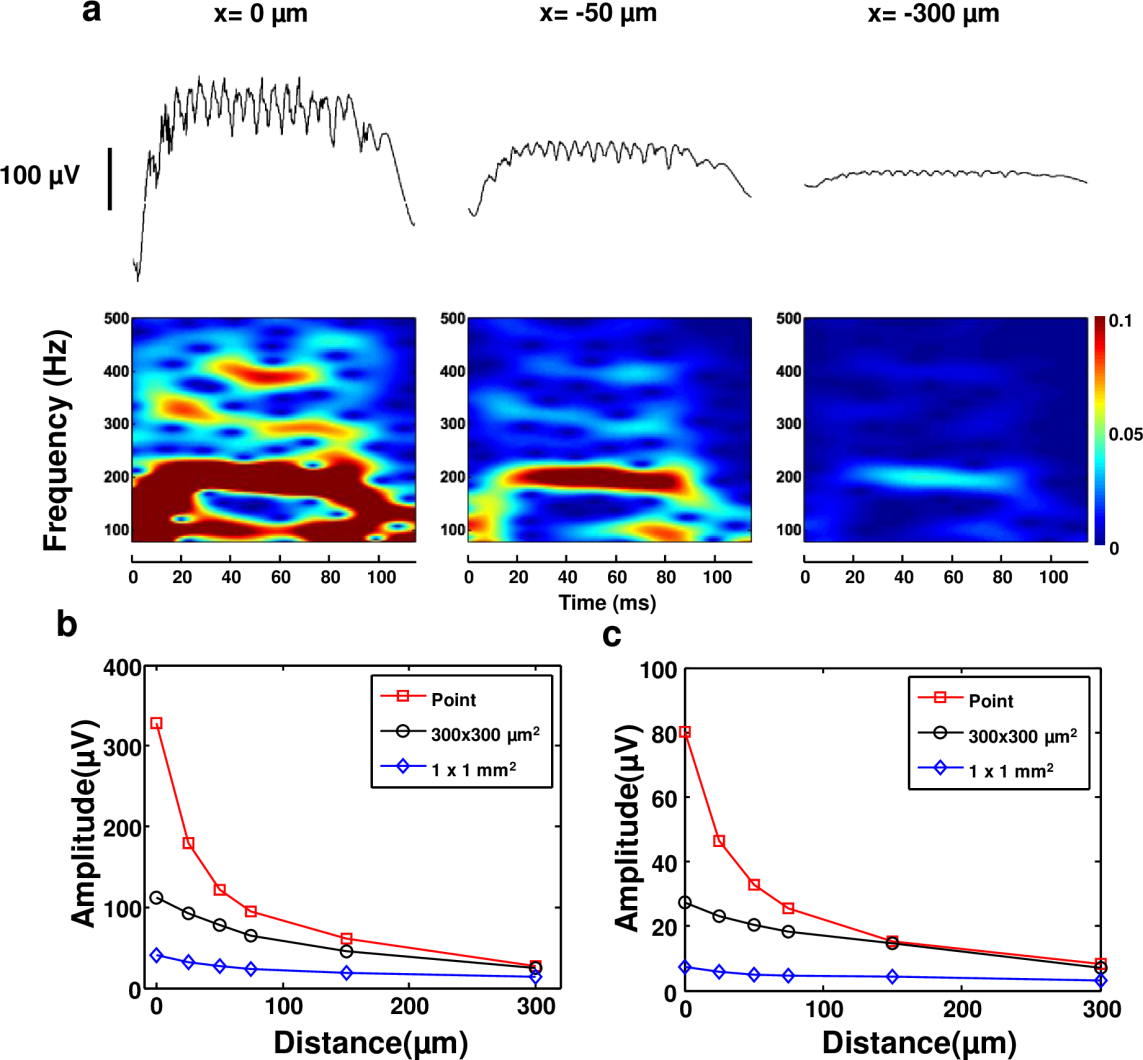
Theoretical analysis of electrode distance. **(a)** Figure shows SPW-R waveforms and spectrograms calculated at 0 μm, 50 μm, and 300 μm distances from the network for point electrodes. **(b)** Amplitude of sharp waves is plotted as a function of distance for three different sizes of electrodes: point, 300 × 300 μm^2^, and 1 × 1 mm^2^. Sharp wave amplitude decreases significantly with increasing distance. **(c)** Amplitude of ripples is plotted as a function of distance. Ripple amplitude decreases with increasing distance.

Fig 4A shows sharp wave ripples calculated for three different distances for an ideal point electrode (See S4 Fig for SPW-Rs calculated at six different distances). The power of the ripples at 200 Hz decreases significantly as the point electrode is moved away from the network. In addition, the power of the oscillations at higher frequencies gets weaker by increasing the distance as a result of 1/r scaling. For instance, at close proximity to the network (0 μm, Fig 4A, left), field potentials exhibit strong oscillation patterns at 100 Hz, 200 Hz, 300 Hz, and even 400 Hz. However, at a distance of 50 μm or farther, higher frequency oscillations diminish and become undetectable at a 300 μm distance. The overall amplitude of the LFPs, including low and high frequency components, decreases notably as a result of the 1/r dependency of potentials generated by current sources.

Fig 4B and 4C shows simulation results for SPW-Rs as a function of distance for three different recording areas (see S5 and S6 Figs for detailed LFP traces and spectrograms recorded by 300 × 300 μm^2^ and 1 × 1 mm^2^ surface electrodes, respectively). The amplitudes of both sharp waves and ripples are found to decrease significantly with increasing distance. The amplitude of ripples is roughly one fourth of the amplitude of sharp-waves. The spatial reach of LFPs for SPW-Rs is found to be around 250–300 μm. Simulations do not exhibit any low or high frequency waveforms beyond 300 μm. The steep decrease of ripple amplitude with distance observed in our simulations is consistent with experimental data[40]. SPW-Rs become less informative when the distance of the recording electrode increases. Our results suggest that for a given distance, a smaller electrode area provides a larger amplitude signal and this effect is more pronounced in close proximity to the network. In addition, these results suggest that ephaptic effects need to be considered only for short distance propagation due to a fast decay with distance of SPW-Rs. Understanding the spatial extent of SPW-Rs from a spatially confined active population may be important for incorporating ephaptic mechanisms for SPW-R propagation across different microcircuits.

### Effect of recording area on SPW-Rs

In order to understand SPW-Rs recorded at different spatial scales, we have studied electrode size dependence of field potential recordings. In experimental studies, SPW-Rs are detected by metal microelectrodes, which have a finite conductive recording surface from a certain distance. We simulated LFPs across a finite-sized planar electrode as described in the *Biophysical Model* section. LFPs are generated by 80 pyramidal cells, 20 basket cells, all of which were active, and 3000 non-spiking pyramidal cells. Surface LFPs are calculated for two different distances from the network, i.e. -75 μm and 0 μm. Six different sizes of surface electrodes (point, 100 × 100 μm^2^, 300 × 300 μm^2^, 1 × 1 mm^2^, 2 × 2 mm^2^, 4 × 4 mm^2^) are simulated. As explained in the *Biophysical Model* section, our calculations give an approximate solution for LFPs recorded across a surface. We investigated how the amplitude and frequency content of SPW-Rs change with the recording area.

**Fig 5:**
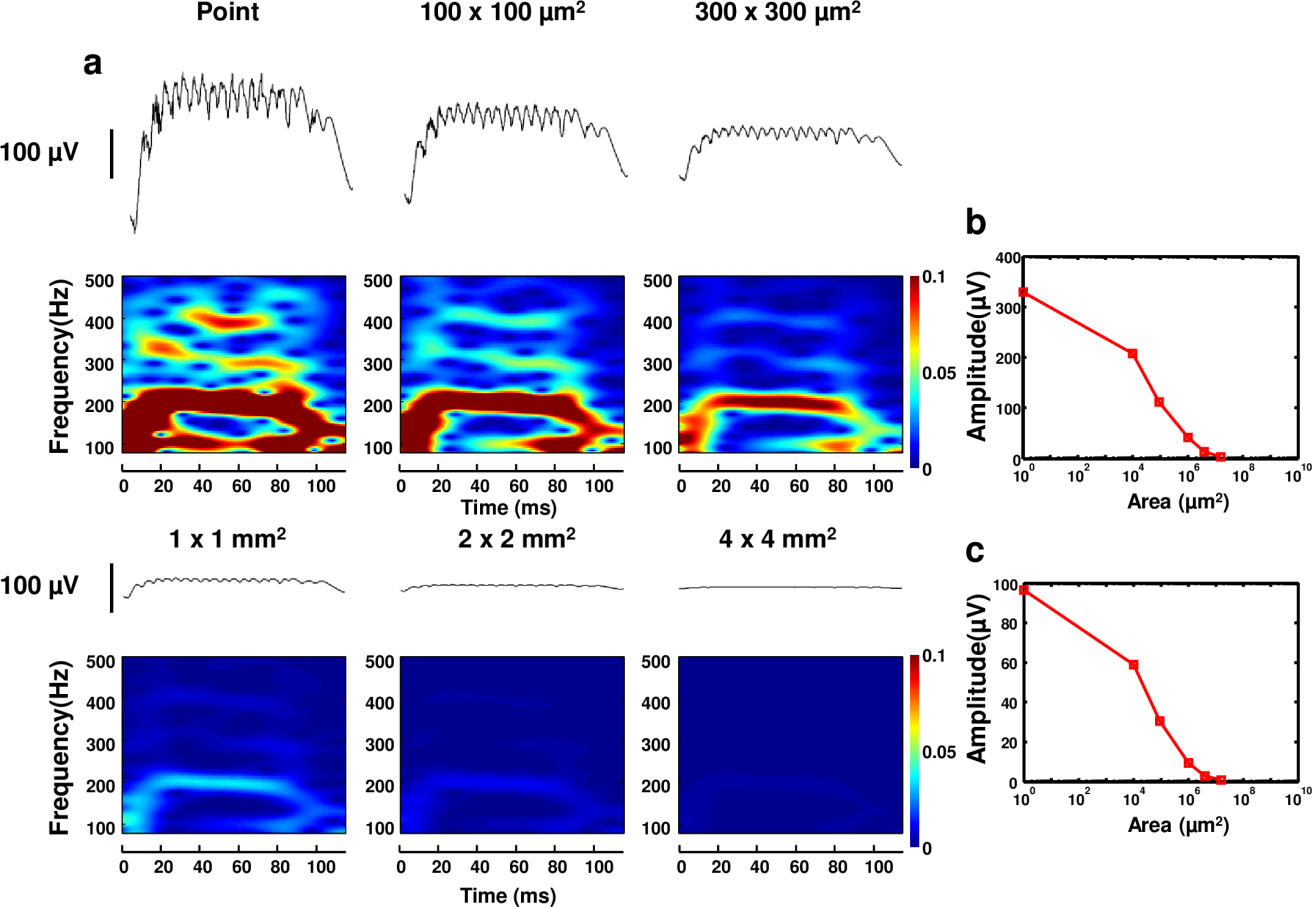
Theoretical analysis of electrode size. Figure shows SPW-Rs computed for different size electrodes (point, 100 × 100 μm^2^, 300 × 300 μm^2^, 1 × 1 mm^2^, 2 × 2 mm^2^, and 4 × 4 mm^2^). It indicates that high frequency oscillations and amplitudes of SPW-Rs decrease significantly with increasing surface area of the recording electrode. **(b)** Amplitude of sharp waves is plotted as a function of electrode area. **(c)** Amplitude of ripples is plotted as a function of electrode area.

Fig 5A displays the spectrograms of LFPs calculated for different areas at 0 μm distance (see S7 Fig for a 75 μm distance and S8 Fig for a comparison between 0 μm and 75 μm in terms of recorded LFP amplitude (μV)). The spectrograms clearly show contributions from spikes to high frequency bands (300 Hz, 400 Hz) when local fields are sampled from an area comparable to the area of active pyramidal neurons (400 × 400 μm^2^). One possible explanation for this is the network structure used in our simulations. The point, 100 × 100 μm^2^ and 300 × 300 μm^2^ electrodes are smaller than the active pyramidal network (400 × 400 μm^2^), and hence they receive strong LFP contributions leading to high amplitude, high frequency oscillations. Spectrograms in Fig 5A show that high frequency oscillations can only be observed for electrodes smaller than 300 × 300 μm^2^. Traces for 1 × 1 mm^2^, 2 × 2 mm^2^ and 4 × 4 mm^2^ in Fig 5 demonstrate that electrode areas larger than the network size (1 × 1 mm^2^) show dramatic decreases in amplitude and power for high frequency oscillations. As plotted in Fig 5B and Fig 5C, the amplitudes of the sharp waves and the ripples exhibit a consistent decrease as a function of electrode size at a 0 μm distance. S8 Fig shows that the relative amplitude decrease is less pronounced at a 75 μm distance. Our findings show that the signal decreases strongly with increasing area and the relative decrease is more pronounced when the recording electrode is closer to the signal source. These findings are consistent with those of Moulin et al. [33] and Moffitt et al. [41].

The results illustrate that underlying network structure and recording configuration are significant factors in determining the electrode size and geometry for measuring high amplitude LFPs. In the previous section, we have already shown that the LFP amplitude is inversely proportional to the distance of the electrodes and that the high frequency contents of LFPs decrease with increasing distance. As the point-source approximation (Eq 1) suggests, extracellular potential Φ will be zero infinitely far away from the transmembrane current [31] due to inverse proportion. With the extension of electrode area, we include points, which are relatively distant from the network contributing almost zero potential to the measurement. Since the LFPs are calculated by including these distant points, spatial averaging results in a decrease in relative amplitude of the signals of interest with increasing electrode area.

Understanding the filtering and attenuation of high frequency signals in neural recordings has always been an interest of the electrophysiology community. The frequency filtering properties of LFPs have some physical grounds, such as Maxwell’s theory, detailed in several studies [42]. Maxwell’s equations state that conductivity and permittivity are one of the two primary determinants of the electric properties of a conductive medium [43]. In the case of non-homogenous extracellular structure, Bédard et al. has already shown the origin of the frequency-dependent attenuation [42]. They found that spikes recorded close to the soma contain more powerful signals in higher frequencies compared with the spikes recorded far away from the soma [42,44]. They also stated that the low-pass filtering effect cannot arise for a homogenous extracellular medium [42,44]. Contrary to this idea, Pettersen and Einevoll showed that this result can be applicable even if the extracellular medium is homogenous [44]. They found that the spike width increase with increasing soma distance results in a low-pass filtering effect. While recordings close to a somatic point current indicated amplified high-frequency contents, when moving away from soma, dendritic currents become important, and for larger distances, high frequencies get attenuated significantly [44].

While there is still some controversy in understanding the exact cause of these observed phenomena, it is crucial to differentiate attenuation from frequency filtering. Attenuation is defined as a reduction of amplitude of a signal of interest, while filtering means removal of specific frequency content from the signal. Our findings should be understood in the context of attenuation as a result of the 1/r dependence of field potentials. Both low frequency and high frequency signals attenuate with distance, as shown in Fig 4. Since surface electrodes report the average voltage of all point electrodes, we observe a relative loss in the amplitude of sharp waves and ripples as a result of a larger recording surface consistent with experimental observations. Due to the fact that ripples have much smaller peak-to-peak amplitude than sharp waves, they decrease below detection limits before the sharp waves. According to the spectrograms shown in Fig 5, ripples have not been observed for electrodes with a surface size greater than 300 × 300 μm^2^.

These explanations suggest that amplitudes of both sharp waves and ripples decrease with increasing area, emphasizing the importance of capturing microcircuit activity with fine spatial resolution. This result is consistent with previous experimental findings, which show that high frequency oscillations are detected more accurately using higher resolution [45,46].

### SPW-Rs computed across a microelectrode array

Microelectrode arrays (MEAs) have been widely utilized to detect neuronal signals *in vitro* and *in vivo* [47]. MEAs are capable of multisite, parallel recording and simultaneous stimulation of neurons at multiple sources and they can provide statistical results in a short period of time [47]. SPW-Rs have also been experimentally investigated using microelectrode arrays (MEAs) with hippocampal slice preparations [48–50]. Here, we have simulated a microelectrode array that spans an area of 4 × 4 mm^2^ using the network described in the *Biophysical Model* section. The size and spacing of the electrodes in MEAs can vary considerably depending on the experimental needs or specific research application. Our array consists of 65 electrodes, all of which have an area of 100 × 100 μm^2^. LFP calculation for each surface was done using Eq 2. Except for the electrode at the center of the array, all the electrodes are spaced 500 μm laterally and 500 μm horizontally starting from -1750 μm to 1750 μm. Eighty pyramidal and 20 basket cells were active while 3000 pyramidal cells were inactive. We have placed an extra electrode at the center of the network to be consistent with the simulations in the previous sections. In these simulations, we used synchronous pyramidal cell spiking as the mechanisms to generate SPW-Rs.

**Fig 6:**
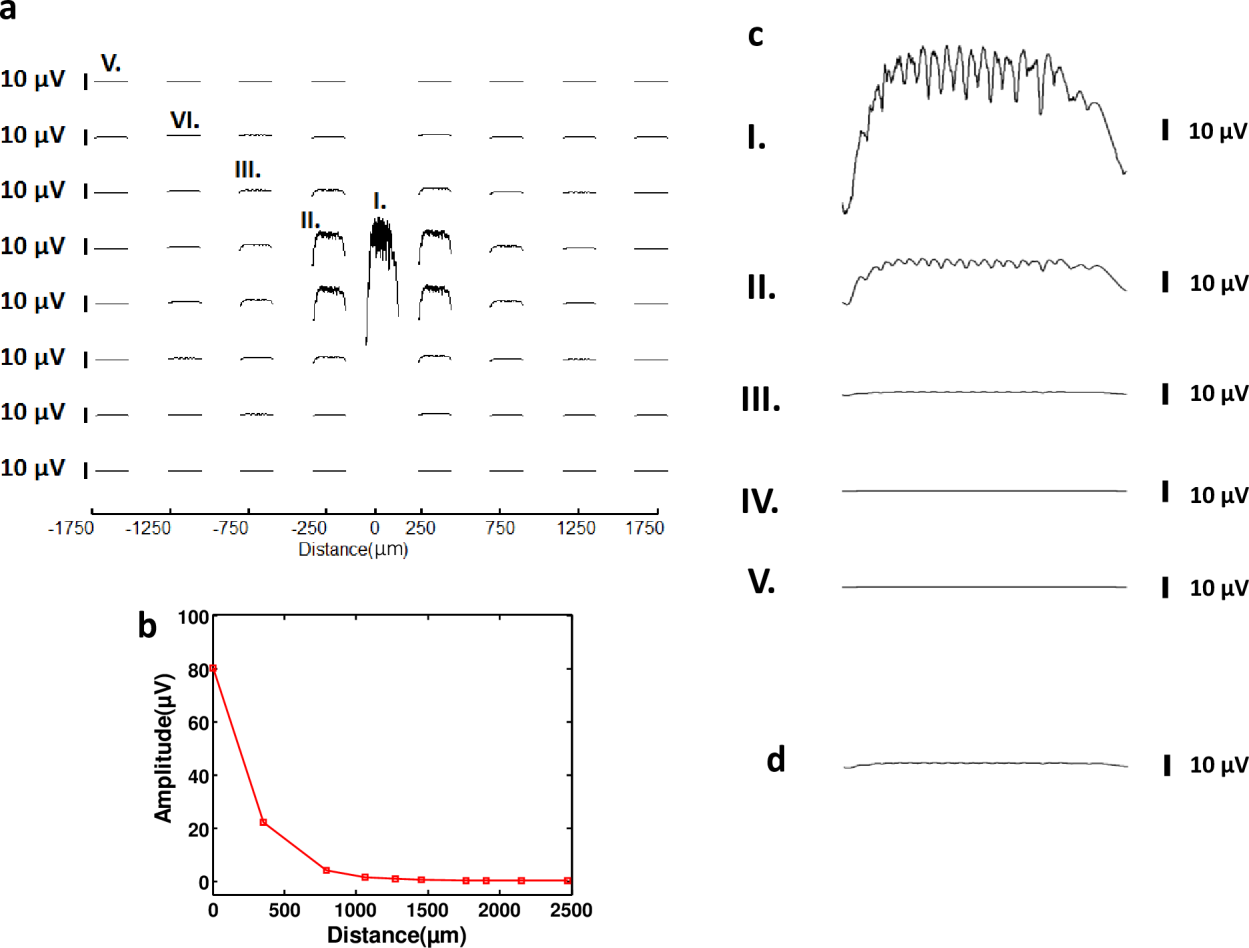
Microelectrode simulation was performed to observe the recorded SPW-Rs in different regions of neural network. **(a)** SPW-R traces computed for a 65-electrode multi electrode array which spans a 3.5 × 3.5 mm^2^ total area (-1750 μm to 1750 μm). Electrodes are 100 × 100 μm^2^ with 500 μm inter-electrode spacing. One extra electrode is placed at the center of the network. **(b)** SPW-R trace calculated using a 4 × 4 mm^2^ electrode, comparable to clinical electrodes. (**c)** SPW-R traces calculated at different sites of MEA. **(d)** SPW-R amplitude recording as a function of distance, revealing that LFP amplitudes show a decrease as a function of electrode distance to the network.

The goal of this study was to observe the SPW-R measurements in different regions of the network while providing synaptic noise to an active pyramidal cell population in a large scale network. The simulation results show that for electrodes farther away than ~350 μm, the recorded SPW-Rs are very close to zero (Fig 6A. (see Fig 6C for the magnified version of Fig 6A)). The electrode at the center has measured an amplitude of 80 μV, which is considerably higher compared to the SPW-R recordings of the other electrodes in the array. One possible explanation of this fact is the positioning of the electrode with respect to active pyramidal and basket cells. All of the electrodes except for the one at the center were located off the axis from the active network. Therefore, the synaptic contributions originating from these cells remained limited, producing substantial differences in the SPW-R amplitude. The SPW-R amplitude dependence on distance was explained in the *Effect of Distance on SPW-Rs* section in detail. The electrodes at a distance around 350 μm have recorded low amplitude signals, ~20 μV, which indicates the significance of contributions coming from inactive pyramidal cells. It should be also pointed out that there is a symmetry in SPW-R traces, as depicted in Fig 6A. The electrodes located at the same absolute distance from the center resulted in similar spike patterns, which is also consistent with Eq 1 (see S9 Fig for the spatial difference of SPW-Rs recorded by four electrodes located with same absolute distance). Minor spatiotemporal differences were observed depending on the coordinate of the recording site relative to the network (S9B Fig).

We have also compared MEA simulations with a hypothetical mm-scale electrode, with an area of 4 × 4 mm^2^, comparable to the size of conventional clinical electrodes. As shown in Fig 6D, the mm-scale electrode recordings do not exhibit any measureable oscillations. The SPW-R is also very close to zero as depicted in Fig 6D. These findings suggest that the spatial resolution of the LFP recordings is a key factor to probe sparse microcircuit activity and high frequency oscillations.

## Discussion

Our findings suggest that SPW-R amplitude and measurable frequency content can vary significantly depending on the electrode resolution and positioning. Therefore, it is necessary to select an appropriate electrode size and position to perform high sensitivity SPW-R recordings. Simulation results, also supported by theoretical calculations, illustrate that the amplitude of sharp waves and ripples exhibit a fast decay with an increasing distance between the biophysical network and the recording electrode. In this study, we investigated distances in the range of 0 μm to 300 μm with respect to the center of the network. For distance values greater than ~300 μm, the simulation results do not exhibit measurable oscillations. Analyzing spectrograms for different distances, it is observed that strong oscillation patterns at 200 Hz, 300 Hz, and 400 Hz can only be detected if the electrode is located in close proximity to the network.

Detection of high frequency oscillations (HFOs) in experimental settings highly depends on the size of the recording electrodes, recording configuration and their positioning with respect to cellular layers [51]. In addition, the sources generating HFOs (pathologic vs. transient) can significantly affect their spatial spread and the volume of the tissue contributing to these oscillations. Therefore, experimental results on detection of HFOs with macro- or microelectrodes suggest different views on the subject. Experimental studies on transient HFOs suggest that macroelectrode and microelectrode recordings exhibit different spectral features [46]. Spectral power of HFOs is shifted to higher frequency bands for the microelectrode recordings than the recordings with macroelectrodes. In contrast, some pathological studies with different electrode sizes do not show any difference in HFO characteristics with respect to the electrode area [52,53]. Discrepancies among these studies could be attributed to the specific positioning of the electrodes with respect to cellular layers, the distance between HFO generating sites and the electrode and the volume of the tissue generating HFOs [51]. Recordings from different layers of the hippocampus show differences in spectral frequency content and amplitude [54], which might also explain some of the discrepancies observed in experimental studies.

While simulations of LFPs for point electrodes have a strong theoretical background [55], modeling of LFPs recorded by electrodes with finite surface areas is more complicated. To-date various different techniques have been employed in the literature. We have investigated the effect of electrode size on SPW-R recordings by computing the local field potentials across the electrode surface. LFPs were calculated using superposition of potentials generated by each compartment of the neurons using Eq 1 [55]. The average potential across the surface was computed by averaging potentials across the electrode surface using Eq 2. Although averaging has been used in this study and several other studies in the literature [31,33,34] to calculate potentials detected by electrodes with a finite surface area, it is important to emphasize that this method does not include higher order electric field or edge effects, depending on electrode geometry. A more complete and detailed treatment could utilize frequency-dependent finite element models for the microelectrodes [35] coupled with the neuronal network model. Surface averaging used in this study only gives a reasonable approximation to computationally demanding FEM analysis [34].

Our simulations on the effect of the electrode size suggest that increases in the area of electrodes results in a drastic decrease in SPW-R amplitude. We detected lower SPW-R amplitudes as we extended the surface, since farther points are included in the integration for calculation of the LFPs. The primary reason of why we observe amplitude decrease as we extend the electrode surface is the 1/r dependence of the extracellular potential, which is implied by Eq 1. The spatial reach of the LFP around a recording contact is another key factor to determine LFP amplitude, as addressed in several studies [35,56]. Therefore, choosing an electrode size comparable to the size of the active neuron population, i.e., basket cells and pyramidal cells, is critical to detect SPW-Rs with high sensitivity. It should be noted that the high frequency ripples were attenuated below experimentally measurable limits for large recording contacts, i.e. 300 × 300 μm^2^, 1 × 1 mm^2^, 2 × 2 mm^2^, and 4 × 4 mm^2^. This is a result of the 1/r dependency of field potentials and spatial averaging across the recording area of the electrodes.

Finally, to gain a better insight on how spatiotemporal properties of the SPW-Rs change in 2D space, we calculated LFPs across a 65 electrode MEA. This study suggests that LFP amplitudes are attenuated for the electrodes away from the center of the network. Simultaneous recordings of electrodes also revealed that symmetric electrodes with respect to the origin of the model measured similar frequency content and wave patterns. The SPW-R traces recorded by electrodes located at the same absolute distance were almost identical, which is also compatible with Eqs 1 and 2. Beyond a 350 μm lateral distance from the network center, the LFPs were not detectable since limited contributions from the activated neurons were averaged out with the contributions from the inactive cells.

## Conclusion

In this work, we studied spatiotemporal characteristics of SPW-Rs using a biophysical model of the hippocampus. We investigated the effect of recording electrode size and distance in order to delineate different network and recording configurations. Moreover, we explored two major contributors to SPW-Rs, i.e., neuronal spikes and synaptic potentials, by using two different configurations: synchronous pyramidal cell spiking and synchronous IPSPs. We have shown that different sizes of surface electrodes can differentiate the characteristics of SPW-Rs. Finally, a MEA, which contains 65 electrodes, was simulated to monitor neural signals from different recording sites of our network. Voltage values measured by the electrodes beyond ~350 μm were below measurable limits and also high frequency contents disappeared as electrodes were located farther away. Finally, we made a comparison between a mm-scale electrode and a high density MEA in terms of the spatial scale and power spectrum of recorded SPW-Rs.

## Supporting information

S1 Fig Spike raster of all cells and LFP traces and spectrograms for the parts of the cells in the model. **(a)** Spike raster of all cells. Neuron number from 0 to 79 indicates the active pyramidal cells, neuron number from 80 to 99 indicates the basket cells, neuron number from 99 to 3100 indicates the inactive pyramidal cells. **(b)** LFP contributions from dendrite and soma of active pyramidal cells. **(c)**, **(d)**, **(e)** Total contribution from active pyramidal cells, total contribution from inactive pyramidal cells, and contribution from basket cells are shown, respectively.
(TIF)

S2 Fig Theoretical analysis of electrode distance by using point electrodes for SPW-Rs generated by IPSPs. **(a)** SPW-Rs are calculated at six different distances (0 μm, 5 μm, 25 μm, 50 μm, 75 μm, 150 μm) by using point electrodes. Results for three distances (0 μm, 50 μm, 150 μm) are shown in (a). **(b)** Amplitude of sharp waves at six different distances (0 μm, 5 μm, 25 μm, 50 μm, 75 μm, 150 μm). **(c)** Amplitude of ripples at six different distances (0 μm, 5 μm, 25 μm, 50 μm, 75 μm, 150 μm).
(TIF)

S3 Fig Theoretical analysis of the number of inactive pyramidal cells. SPW-Rs are calculated for the networks with the number of inactive pyramidal cells of 3000 and 10000. Spectrograms of both total SPW-Rs and contributions from IPSPs and SPW-Rs waveforms are shown.
(TIF)

S4 Fig Theoretical analysis of electrode distance by using point electrodes. SPW-Rs are calculated at six different distances (0 μm, 25 μm, 50 μm, 75 μm, 150 μm, 300 μm) by using point electrodes.
(TIF)

S5 Fig Theoretical analysis of electrode distance by using 300 × 300 μm^2^ surface electrode. SPW-Rs are calculated at six different distances (0 μm, 25 μm, 50 μm, 75 μm, 150 μm, 300 μm) by using 300 × 300 μm^2^ surface electrode.
(TIF)

S6 Fig Theoretical analysis of electrode distance by using 1 × 1 mm^2^ surface electrode. SPW-Rs are calculated at six different distances (0 μm, 25 μm, 50 μm, 75 μm,150 μm, 300 μm) by using 1 × 1 mm^2^ surface electrode.
(TIF)

S7 Fig Theoretical analysis of electrode size for distance 75 μm. **(a)** SPW-Rs are recorded by six different size microelectrodes (point, 100 × 100 μm^2^, 300 × 300 μm^2^, 1 × 1 mm^2^, 2 × 2 mm^2^, 4 × 4 mm^2^ for distance 75 μm. **(b)** Amplitude of sharp waves at six different surface area for distance 75 μm. **(c)** Amplitude of ripples as a function of electrode area for distance 75 μm.
(TIF)

S8 Fig Amplitude change of sharp waves and ripples as a function of distance for 0 μm and 75 μm distances. **(a)** Sharp wave amplitudes recorded by six different surface areas (point, 100 × 100 μm^2^, 300 × 300 μm^2^, 1 × 1 mm^2^, 2 × 2 mm^2^, 4 × 4 mm^2^). **(b)** Ripple amplitudes recorded by six different surface areas (point, 100 × 100 μm^2^, 300 × 300 μm^2^, 1 × 1 mm^2^, 2 × 2 mm^2^, 4 × 4 mm^2^).
(TIF)

S9 Fig LFP traces recorded by four closest electrodes with respect to the origin of the biophysical model. **(a)** LFP recordings reveal that there is a symmetricity in LFP traces. The electrodes located with same absolute distance resulted in similar, not exactly same, spike pattern. (II) in Fig 6A refers to the upper-left electrode. **(b)** Magnified version of (a).
(TIF)
