# Supplementary figures and images for: Computational analysis of network activity and spatial reach of sharp wave-ripples
Source: PLoS One. 2017 Sep 15;12(9):e0184542. doi: 10.1371/journal.pone.0184542 (PMC5600383; doi:10.1371/journal.pone.0184542)

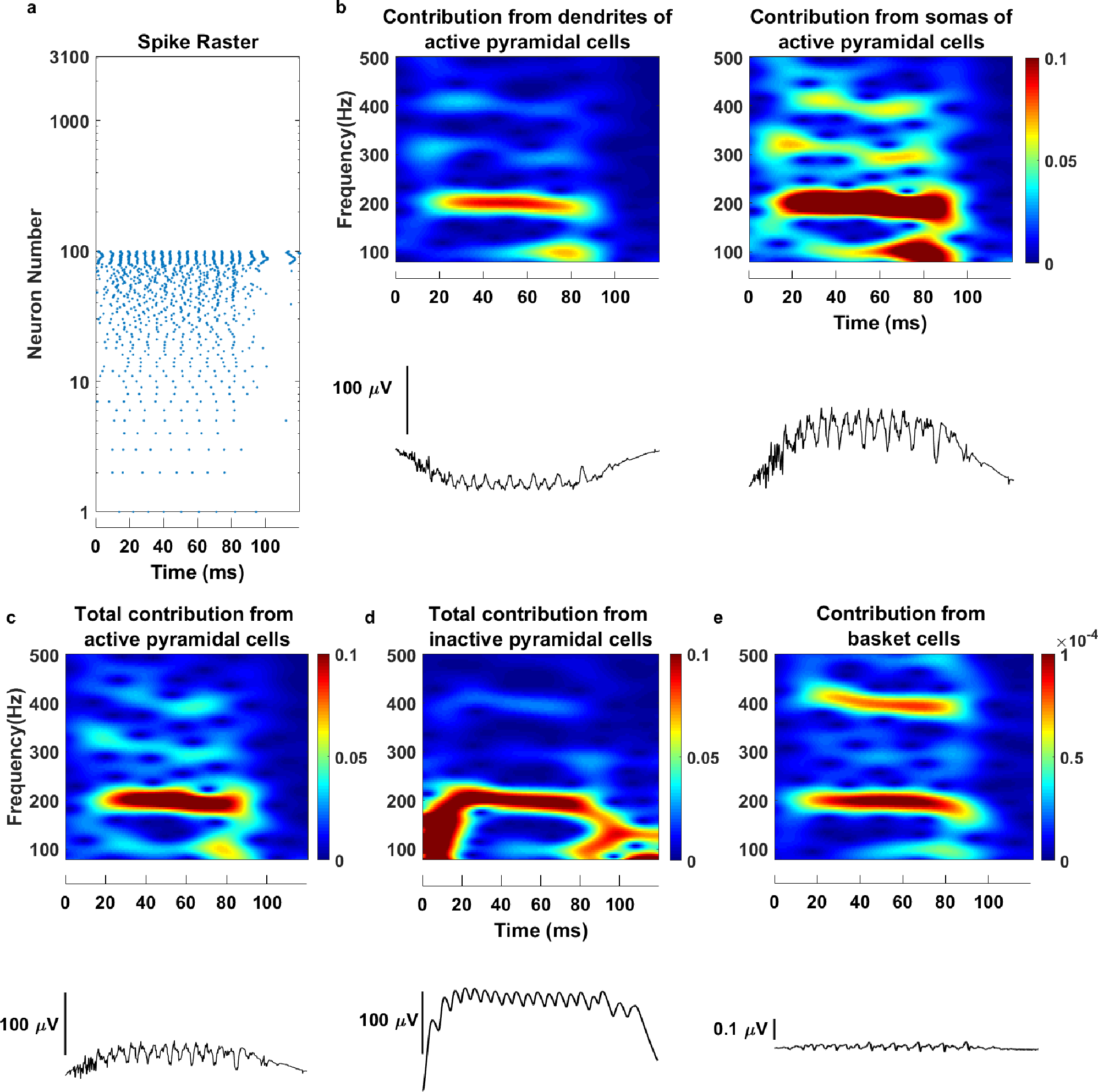

Supplement: S1 Fig — (a) Spike raster of all cells. Neuron number from 0 to 79 indicates the active pyramidal cells, neuron number from 80 to 99 indicates the basket cells, neuron number from 99 to 3100 indicates the inactive pyramidal cells. (b) LFP contributions from dendrite and soma of active pyramidal cells. (c), (d), (e) Total contribution from active pyramidal cells, total contribution from inactive pyramidal cells, and contribution from basket cells are shown, respectively. (TIF) [file pone.0184542.s001.tif]

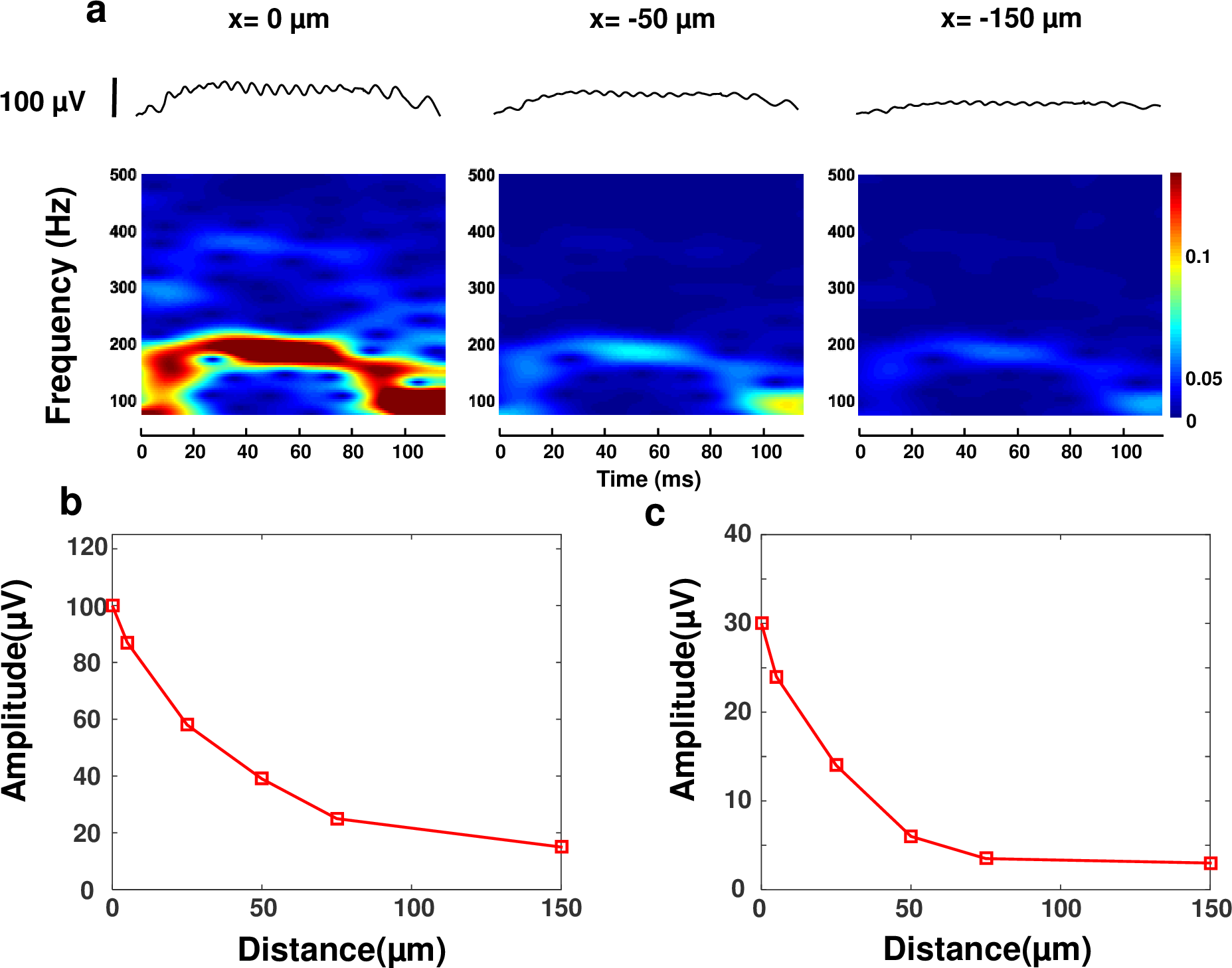

Supplement: S2 Fig — (a) SPW-Rs are calculated at six different distances (0 μm, 5 μm, 25 μm, 50 μm, 75 μm, 150 μm) by using point electrodes. Results for three distances (0 μm, 50 μm, 150 μm) are shown in (a). (b) Amplitude of sharp waves at six different distances (0 μm, 5 μm, 25 μm, 50 μm, 75 μm, 150 μm). (c) Amplitude of ripples at six different distances (0 μm, 5 μm, 25 μm, 50 μm, 75 μm, 150 μm). (TIF) [file pone.0184542.s002.tif]

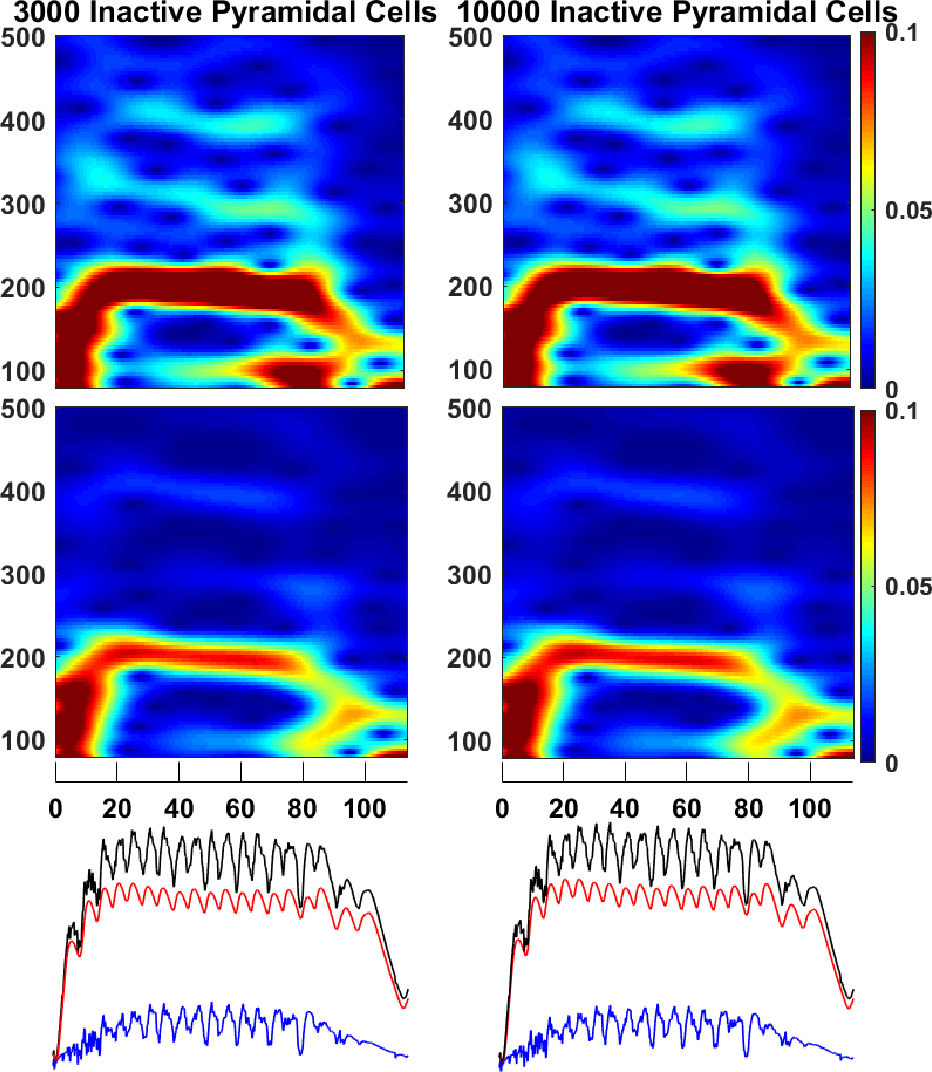

Supplement: S3 Fig — SPW-Rs are calculated for the networks with the number of inactive pyramidal cells of 3000 and 10000. Spectrograms of both total SPW-Rs and contributions from IPSPs and SPW-Rs waveforms are shown. (TIF) [file pone.0184542.s003.tif]

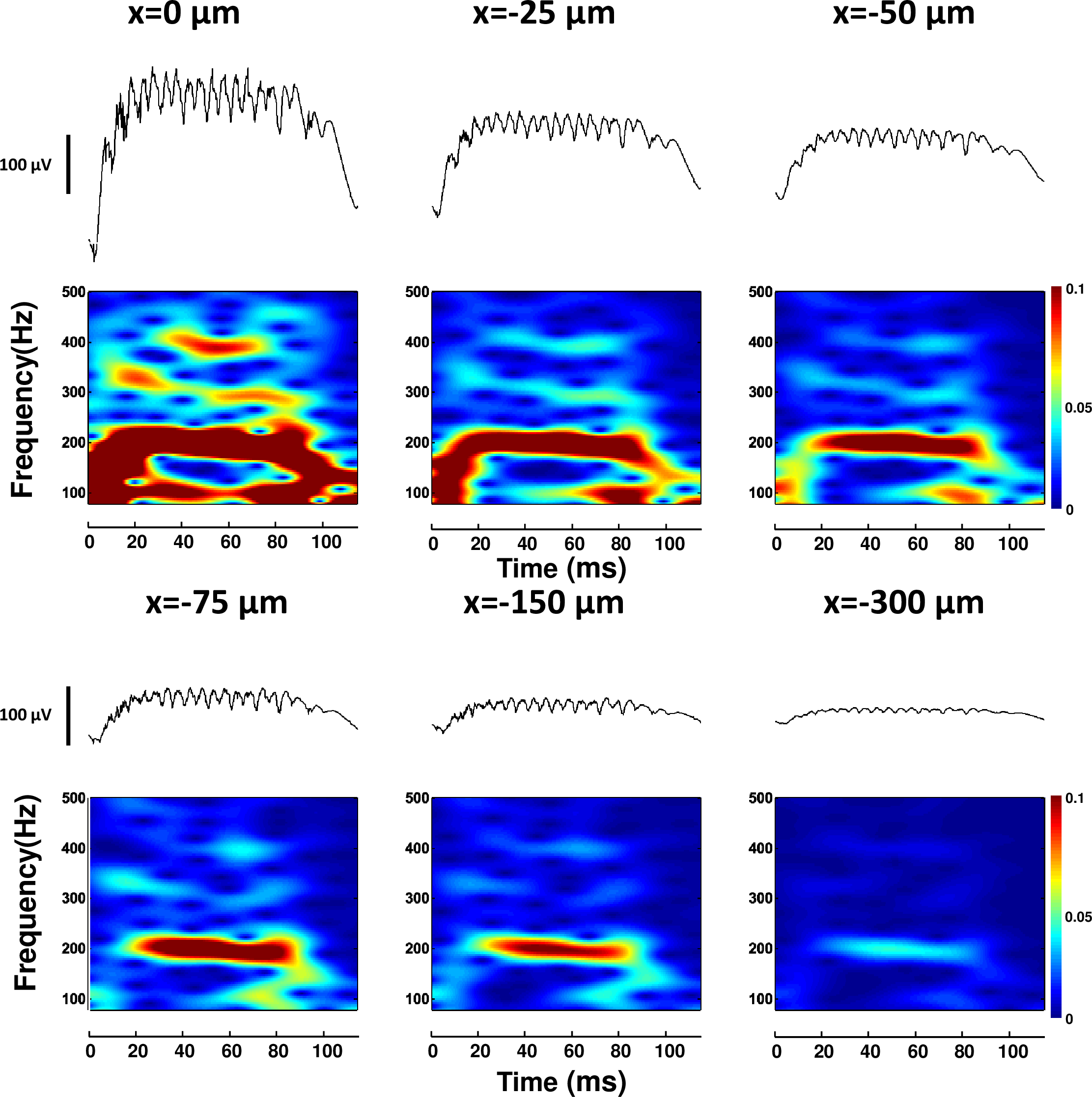

Supplement: S4 Fig — SPW-Rs are calculated at six different distances (0 μm, 25 μm, 50 μm, 75 μm, 150 μm, 300 μm) by using point electrodes. (TIF) [file pone.0184542.s004.tif]

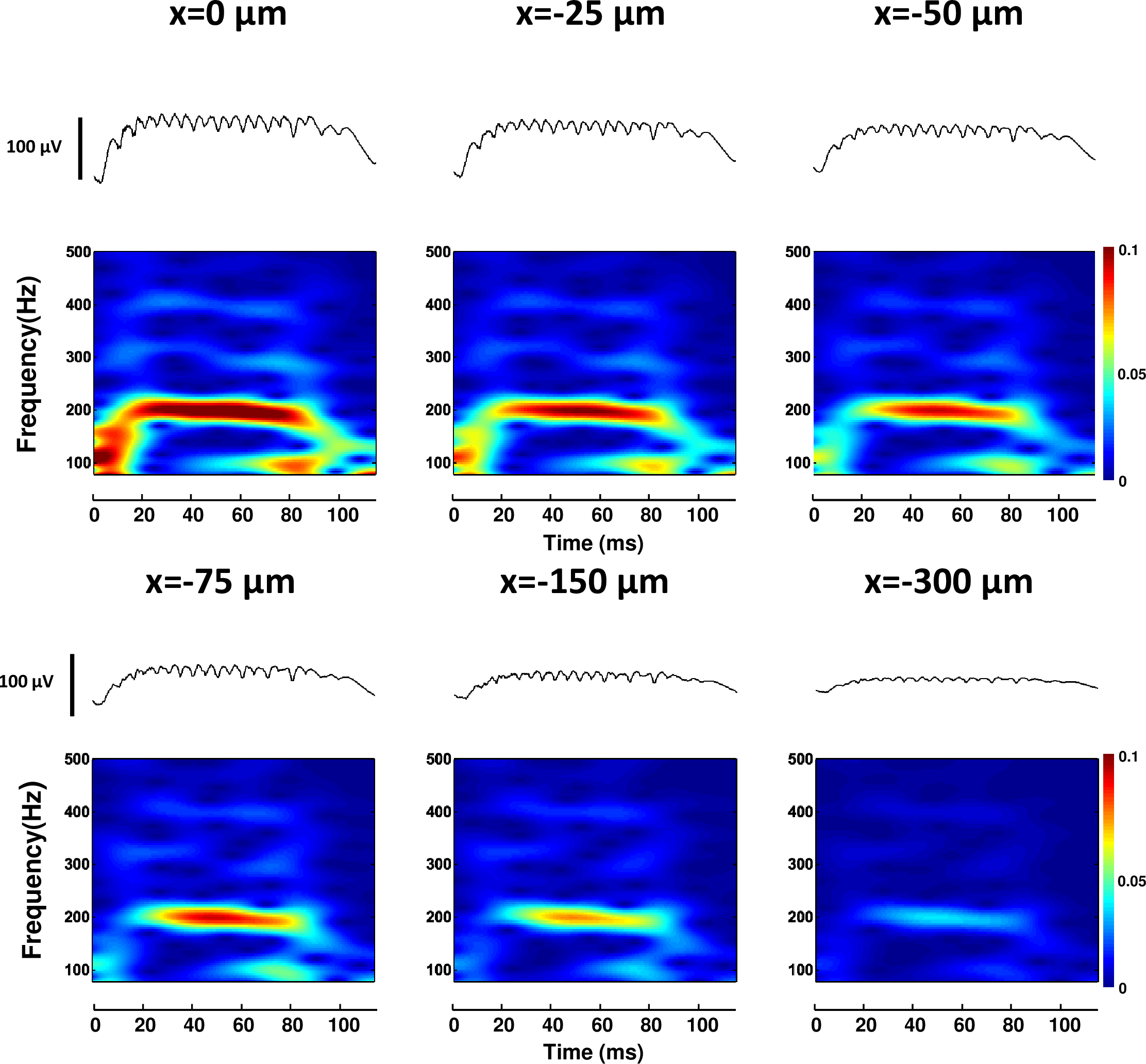

Supplement: S5 Fig — SPW-Rs are calculated at six different distances (0 μm, 25 μm, 50 μm, 75 μm, 150 μm, 300 μm) by using 300 × 300 μm2 surface electrode. (TIF) [file pone.0184542.s005.tif]

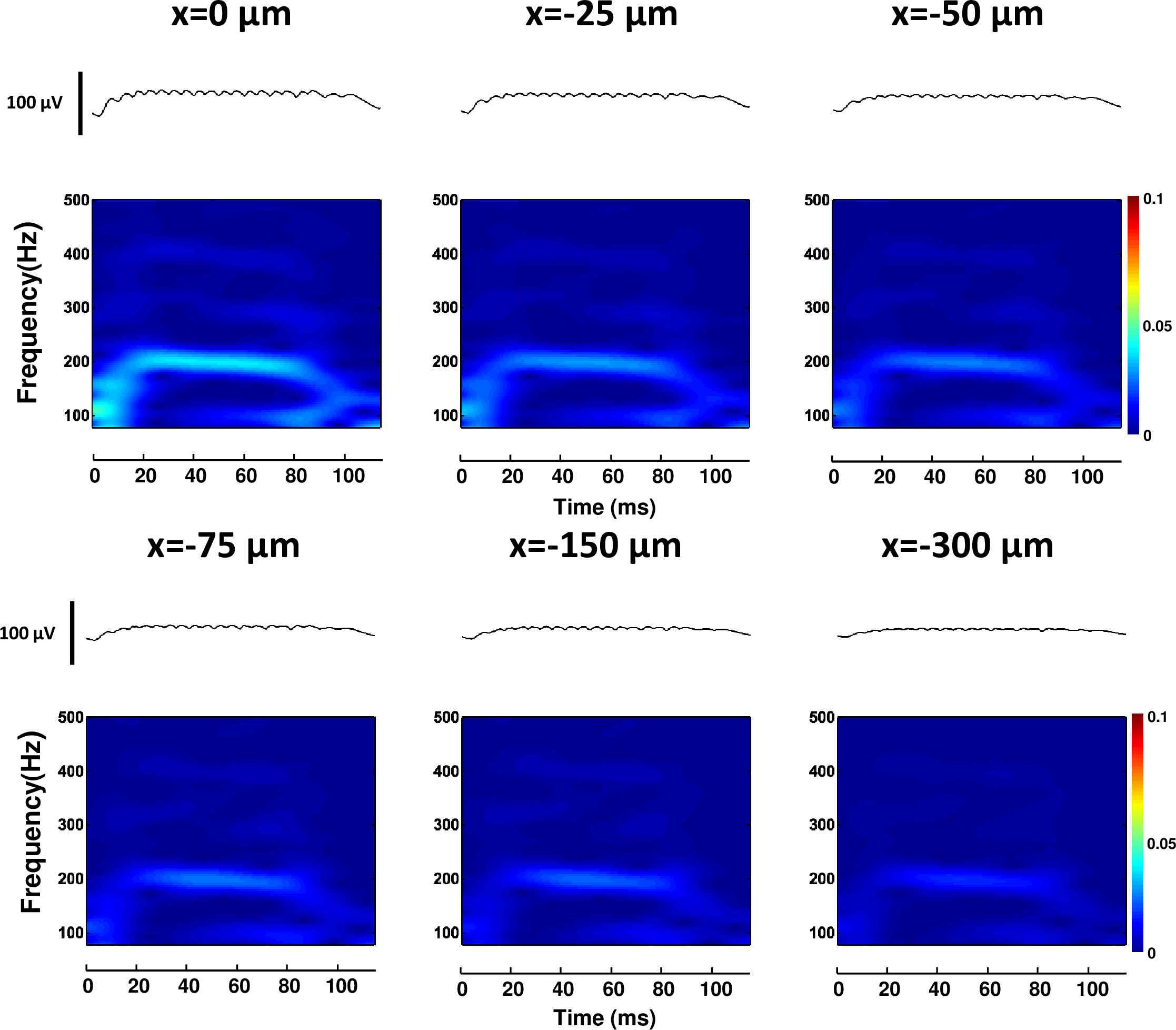

Supplement: S6 Fig — SPW-Rs are calculated at six different distances (0 μm, 25 μm, 50 μm, 75 μm,150 μm, 300 μm) by using 1 × 1 mm2 surface electrode. (TIF) [file pone.0184542.s006.tif]

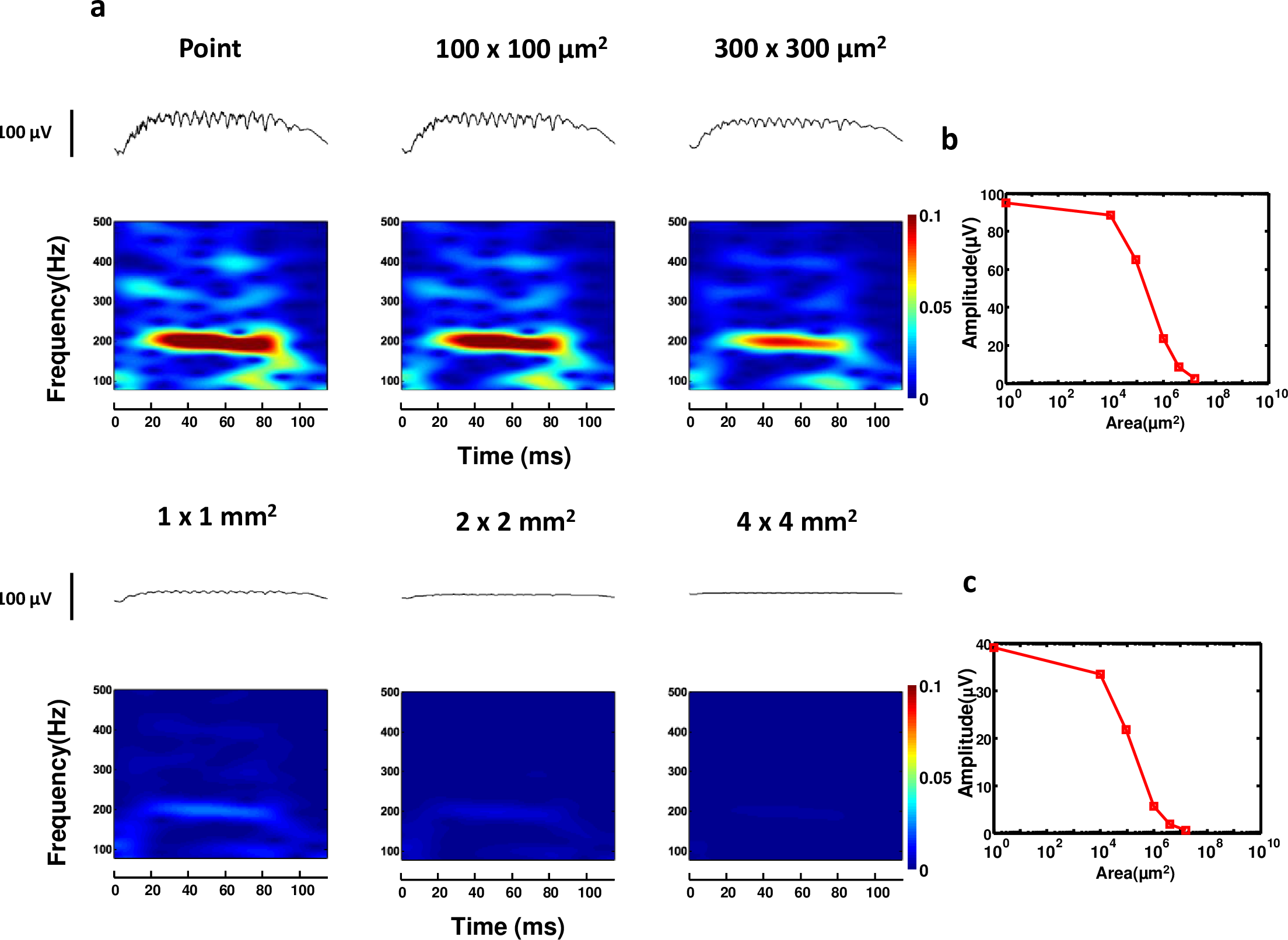

Supplement: S7 Fig — (a) SPW-Rs are recorded by six different size microelectrodes (point, 100 × 100 μm2, 300 × 300 μm2, 1 × 1 mm2, 2 × 2 mm2, 4 × 4 mm2 for distance 75 μm. (b) Amplitude of sharp waves at six different surface area for distance 75 μm. (c) Amplitude of ripples as a function of electrode area for distance 75 μm. (TIF) [file pone.0184542.s007.tif]

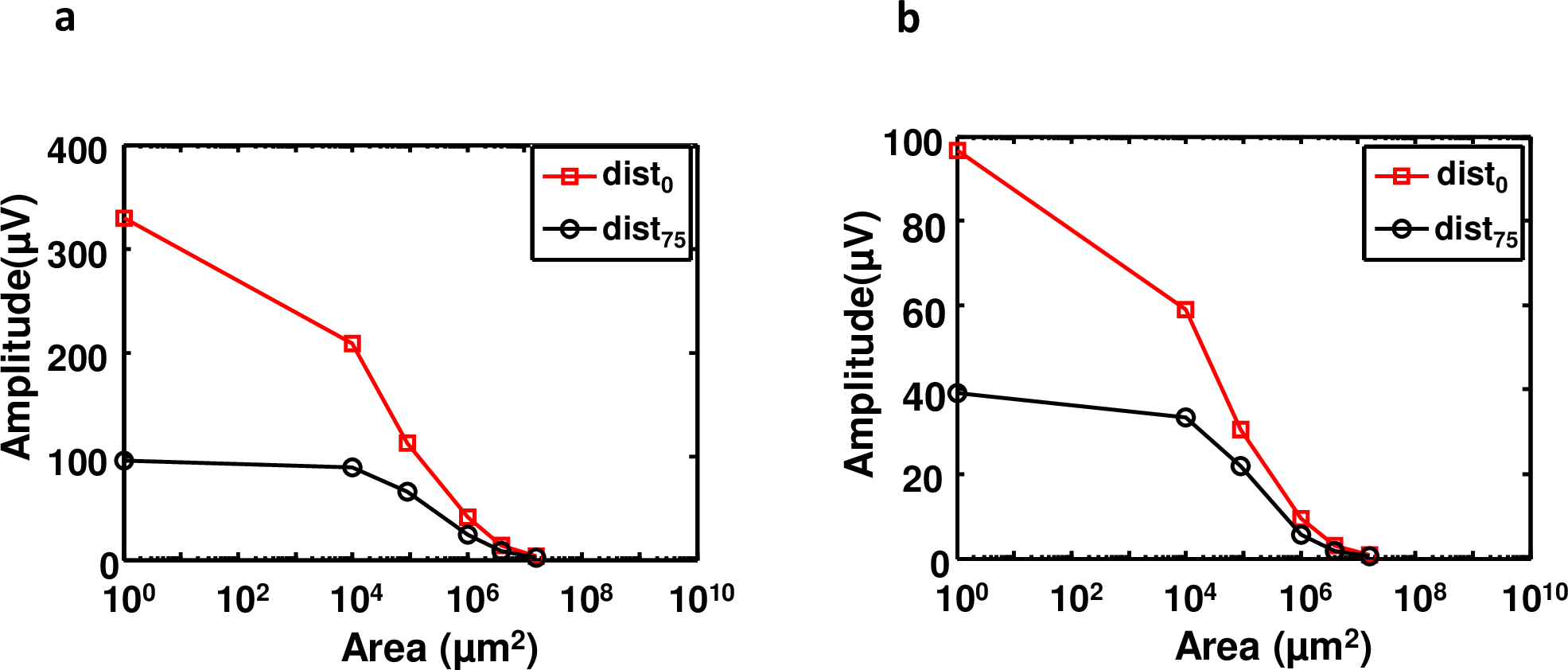

Supplement: S8 Fig — (a) Sharp wave amplitudes recorded by six different surface areas (point, 100 × 100 μm2, 300 × 300 μm2, 1 × 1 mm2, 2 × 2 mm2, 4 × 4 mm2). (b) Ripple amplitudes recorded by six different surface areas (point, 100 × 100 μm2, 300 × 300 μm2, 1 × 1 mm2, 2 × 2 mm2, 4 × 4 mm2). (TIF) [file pone.0184542.s008.tif]

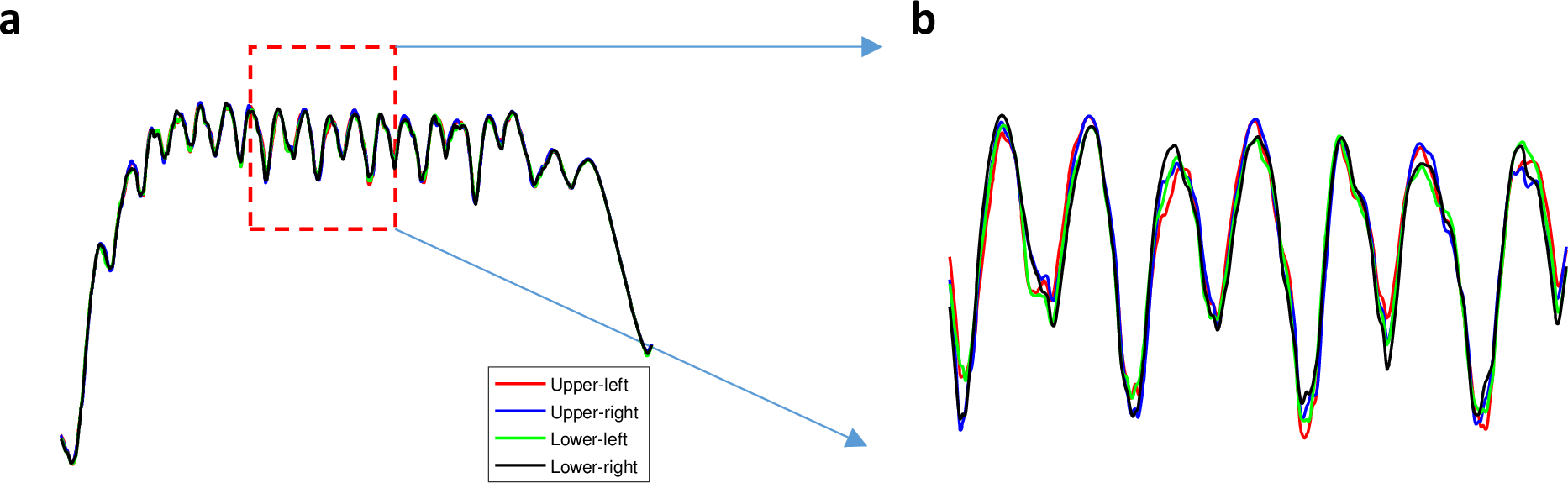

Supplement: S9 Fig — (a) LFP recordings reveal that there is a symmetricity in LFP traces. The electrodes located with same absolute distance resulted in similar, not exactly same, spike pattern. (II) in Fig 6A refers to the upper-left electrode. (b) Magnified version of (a). (TIF) [file pone.0184542.s009.tif]
